# Behavioral and biochemical effects of pharmacopuncture (ST 36 and ST 25) in obese rats

**DOI:** 10.1186/s12906-015-0829-7

**Published:** 2015-08-28

**Authors:** Mariana Chiste Pontes, Lilian Cardoso Heck, Janice Carneiro Coelho

**Affiliations:** Department of Biochemistry, Federal University of Rio Grande do Sul, Rua Ramiro Barcelos, 2600 anexo, 90035-003 Porto Alegre, RS Brazil

**Keywords:** Obesity, Alimentary behavior, Pharmacopuncture

## Abstract

**Background:**

Acupuncture has been reported as a weight loss treatment for obese patients. The use of pharmacopuncture focusing on behavioral analyses has not yet been studied with the objective of treating obesity. Thus, this study aimed to assess the biochemical and behavioral effects of using pharmacopuncture techniques in obese Wistar rats.

**Methods:**

The treatments consisted in applying pharmacopuncture at the Zusanli (ST 36) and Tianshu (ST 25) points.

**Results:**

When treated with pharmacopuncture, groups HDP36 and HDP25 experienced a reduction in body weight compared to the controls, who were also fed a hypercaloric diet. In the alimentary behavior test, latency to feed did not differ between the groups. However, groups HDP36 and HDP25 consumed a smaller number of cereals bits, which suggests that inappetence was an effect of the treatment. No difference was found among the groups in the elevated plus maze test, which indicates no anxiety action of the points studied. Regarding post mortem perirenal and abdominal fat among the groups fed a hypercaloric diet, groups HDP36 and HDP25 had lower perirenal fat weight and HDP36 had lower abdominal fat weight compared to the other groups. Likewise, a reduction in cholesterol 10.1186/s12906-015-0829-7 and glucose levels was found in groups HDP36 and HDP25 compared to the other groups that were fed a hypercaloric diet, while triglycerides decreased in subgroup HDP25

**Conclusions:**

In conclusion, the present study showed the efficacy of pharmacopuncture in weight loss of obese rats, as well as changes in biochemical and behavioral parameters.

## Background

Obesity and overweight are directly linked to the development of a series of chronic disease conditions. The estimated cost for treatment of this chronic disease in the USA is currently over 7 % of all healthcare expenses [10].

Obesity prevalence widely varies among different races and ethnic groups, also being more common in women [31]. Similarly, socioeconomic level and schooling are risk factors of overweight and obesity [25].

Therefore, experts are increasingly interested in studying obesity in order to better understand the factors and processes of this metabolic disorder, proposing solutions to a problem that affects a large portion of the population worldwide, regardless of social class, gender, or age [6].

The changes in lifestyle that lead to a slight weight loss can, in fact, are beneficial to health. The sustained weight loss of 3 to 5 % results in clinically significant reductions in triglycerides, glycemia, and glycated hemoglobin levels, as well as a lowered risk of developing type 2 diabetes [7].

Further weight losses entails a reduction in arterial blood pressure, improved lipid levels, and decreased need for drugs to control blood pressure, glycemia, and lipid levels [11].

The conventional therapy strategies against obesity do not result in appropriate weight control among every treated patient, therefore complementary therapies are also employed [1, 3].

Acupuncture is one of the oldest healing practices and is currently the fastest exponentially growing complementary therapy recognized by the World Health Organization [3].

Pharmacopuncture is also known as acupoint injection or aquapuncture. That means pharmacological medication is injected to acupoints, a new therapy associating acupuncture and medication. New finds reported that pharmacopuncture could provide stronger clinical response than traditional acupuncture [27, 16].

The traditional acupuncture has been reported as a treatment method for weight loss for obese patients [30, 5]. Nevertheless, its mechanisms are still under study and there is no behavioral assessment of the action of pharmacopuncture in obese individuals [28, 3]. Some studies suggest that stimulation by acupuncture may have an effect even in individuals of normal weight [13]. Thus, it is important to investigate which effects occur in individuals of normal weight, in an attempt to clarify the mechanisms involved in stimulation through acupuncture.

Therefore, the present study aimed to assess the biochemical and behavioral effects of pharmacopuncture at acupoints Zusanli (ST 36) and Tianshu (ST 25), as described by the Traditional Chinese Medicine, in obese Wistar rats.

## Methods

Eighty (80) male Wistar rats, with an average weight of 250 ± 10 g were used in the study. The animals came from a monogamous Wistar colony maintained using Poliey heterogeneous breeding method under a controlled conventional sanitary standard at the Bioterium of the Department of Biochemistry of UFRGS.

The “Principles of Laboratory Animal Care” (NIH publication n°85- 2, revised 1985) were followed in all of the experiments and the Ethics Committee for Animal Research of the Federal University of Rio Grande do Sul, Porto Alegre approved the experimental protocol.

The breeders of that colony were kept in a standardized environment in polypropylene cages (414×344×168 mm) with stainless steel lids and a selected autoclaved pine shavings bed under a 12 h light/dark photoperiod (7 AM/7 PM) and controlled temperature (21 °C). The animals used in this study were kept under the same conditions with five animals per cage.

Water and food were provided *ad libitum* for three months prior to the experiment. The animals were split into two groups containing 40 specimens each: Group 1 (control), animals with a balanced diet; and Group 2 (obese), obese animals previously treated with a hypercaloric diet.

The animals in Group 1 were fed water and a balanced diet *ad libitum*. Those in Group 2 were fed a hypercaloric diet (Table 1) throughout the period of the experiment. The animals were kept on this diet for 16 weeks (3 months), which led to a significant weight gain.

**Table 1:** High-calorie diet

Composition	1,000 g
Commercial feed	300
Condensed milk	340
Hydrolyzed soy protein	173
Sucrose	80
Vitamin complex	10
Mineral complex	30
DL-Methionine	3
Lysine	2.5
Soybean oil	60

At first it was necessary to create an animal obesity model in Wistar rats. By using a hypercaloric diet, with excess lipids (vegetable oil) and glycides (condensed milk), a significantly higher weight gain was possible in the animals fed this diet compared to the ones fed the control diet. Battú et al. [2] had already developed this type of model, which aims to increase animal body weight, and the present results match the ones found by those authors.

The two experimental groups were split into subgroups containing ten animals each. They were split in: a group in which the animals received only manipulation, a group in which non –acupuncture points were used in the animals and two groups in which the previously mentioned acupuncture points for the treatment of obesity were utilized. The acupoints used were Zusanli (ST 36) and Tianshu (ST 25). The non-acupuncture point corresponding to a given acupoint was chosen based on its signal being less than half of the conductance measured for that acupoint, laterally to Zuzanli.

In summary, the subgroups, with ten animals each, were as follows: SDH (standard diet/manipulation), SDNO (standard diet non-point), SDP36 (standard diet/point 36), SDP25 (standard diet/point 25), HDH (hypercaloric diet/manipulation), HDNP (hypercaloric diet/non-point), HDP36 (hypercaloric diet/point 36), and HDP25 (hypercaloric diet/point 25).

The intervention was performed once a day for 12 weeks, with the application of bee venom (Sigma-Aldrich®, 0.025 mg/kg) pharmacopuncture, in a concentration of 0.01 mg/0.05 ml. Each animal was administered approximately 0.05 ml, injected subcutaneously on side right of the body. The syringe utilized was 0.45×13, with a needle gauge of 26G× ½”. Body weight was measured prior to and after treatment.

The behavioral parameters were assessed after the acupuncture intervention, using the elevated plus maze test to evaluate anxiety [4] and the alimentary behavior test to analyze motivation for food intake [24].

The elevated plus maze test consists in having the animal move about for five minutes in a cross-shaped device with two open-sided arms and two arms with side walls that provide apparent protection for the animal. This test does not require prior exposure of the animal to the device.

The alimentary behavior test is an analysis of the latency time for the animal to reach a bowl containing 16 units of Froot Loops® with eight different colors, of the latency time until the first bite to the food, and of the number of cereal bits consumed. The device consists of a 50 cm corridor and the animal is placed for three minutes at the opposite end of the bowl with food. This test requires a five-day training period so that the animal can adapt to the device and the new food.

By the end of the pharmacopuncture intervention period, the animals were weighed and euthanized using the guillotine method. Blood and fat tissue samples were collected. Visceral fat (retroperineal and epididymal) was removed and weighed.

Triglycerides, glucose, and cholesterol were assessed using specific commercial kits from Labtest (Brazil) (Triglicérides Liquiform, Glicose PAP Liquiform, and Colesterol Liquiform, respectively) to determine the final reaction point in plasma after it was separated from the blood collected in EDTA tubes by centrifuging at 2.000 rpm for ten minutes. Colorimetric enzymatic assays were used for all analyses, and performed according to the manufacturer’s instructions. The readings were carried out in a UV–Vis spectrophotometer with 505 nm absorbance for triglycerides and glucose, and 500 nm for cholesterol.

The statistical analysis employed ANOVA for the body weight variation, body fat weight, and biochemical analyses, while the Kruskal-Wallis test was used for the behavioral analyses, with a 5 % level of significance.

## Results and discussion

There are several hurdles in the treatment of obesity; a disease that has been continuously growing in the modern world. Among those, it is worth mentioning the lack of adherence to the conventional treatment with diets and physical exercise, as well as the adverse effects of the pharmacological treatment. Acupuncture is a treatment that is being used as a complementary therapy for obesity [3] and is recognized by the WHO. Just the same, it has been shown that pharmacopuncture can be employed more quickly and conveniently than the original technique [23]. No reports were found in the literature on the contribution of bee venom pharmacopuncture for the treatment of obesity in rats. However, there is a study in humans using non related acupoints to this study [21, 22]. Therefore, discussing the results of the effects of pharmacopuncture in points ST 36 and ST 25 becomes crucial, based on the results of the present study.

## Obesity model

**Fig. 1:**
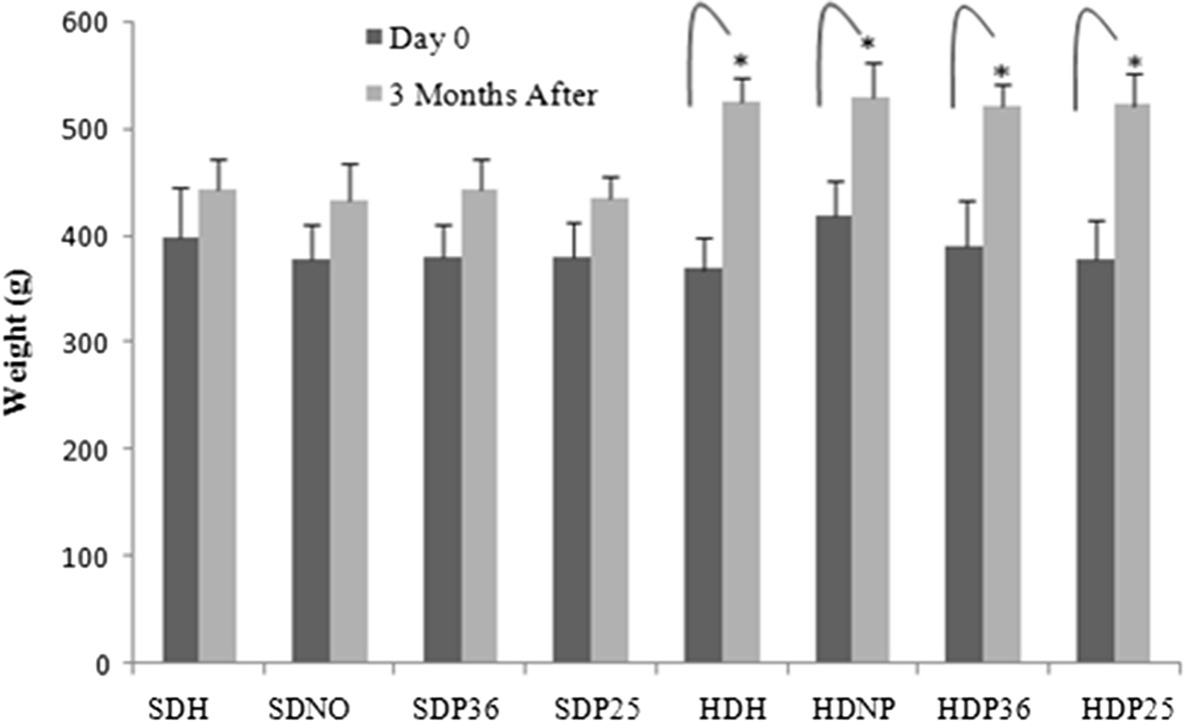
Variation in body weight during the weight-gain period. *Significant (*p* < 0.001) difference when comparing the hypercaloric and standard diet groups

In the three months during which the animals were only fed the diet provided by us, a significant (*p* < 0.001) weight gain was observed in those fed the hypercaloric diet, both in comparison with their initial weight and with the animals that were fed the standard commercial diet (Fig. 1). Both groups gained a significant amount of weight (*p* < 0.001) over the course of treatment (14.3 % for the controls and 34.96 % for the experimental animals), and the experimental (obese) group weighed 19.60 % more than the controls (*p* < 0.001) by the end of the treatment.

From the results obtained, we can see that the hyperpalatable and hypercaloric diet employed in the present study may be widely used as a model of simple obesity. It enabled a 30 to 40 % body weight gain compared to the control diet, as it has been described for other hypercaloric diets [9, 15].

## Variation in body weight

After the model was defined, pharmacopuncture was used in points ST 36 and ST 25, which had never been tested with pharmacopuncture before, only with acupuncture and electroacupuncture.

**Fig. 2:**
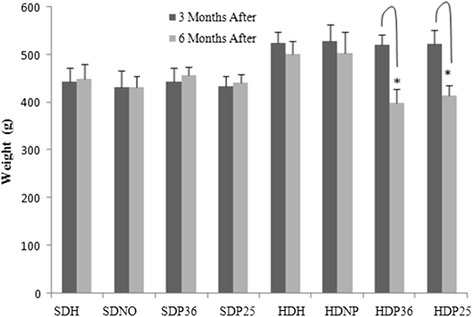
Variation in body weight during the treatment period. *Subgroups HDP36 and HDP25 had a significant (*p* < 0.001) body weight loss

Next, the diet was maintained for all groups and acupuncture interventions were performed for three months. No significant difference was found in the groups fed the standard diet during the course of treatment. However, among the groups fed the hypercaloric diet, a significant (*p* < 0.001) weight loss was observed in groups HDP36 and HDP25, which were treated with acupuncture (Fig. 2).

The positive outcomes of body weight loss from using pharmacopuncture found in the present study are in accordance with those obtained with the use of electroacupuncture and acupuncture in points ST 36 and ST 25 by several authors [17, 28, 29, 14, 32, 15, 20, 19, 8, 33, 12]. However, the method of injection in the acupoint had not been described yet for this type of disorder. Pharmacopuncture has shown to be a promising option, both in the experimental approach, given the easy management, and in the medical and veterinary clinical practice, once it requires shorter treatment time in the office.

We found that pharmacopuncture did not affect weight loss, biochemical or behavioral changes in the animals fed the standard diet, which suggests the need for a metabolic disorder so that a significant result is obtained by using the traditional Chinese medicine technique. According to Shiraishi et al. [26], the results in humans depend on the BMI. Nevertheless, this contrasts with the results by Kim et al. [13] and Kim et al. [14], which report a reduction in body weight by using electroacupuncture even in non-obese individuals.

## Behavioral assessment

These results may target future studies on neurobehavioral analysis of using pharmacopuncture in points ST 36 and ST 25 in the treatment of diet-induced obesity in rats.

### Alimentary behavior test

**Fig. 3:**
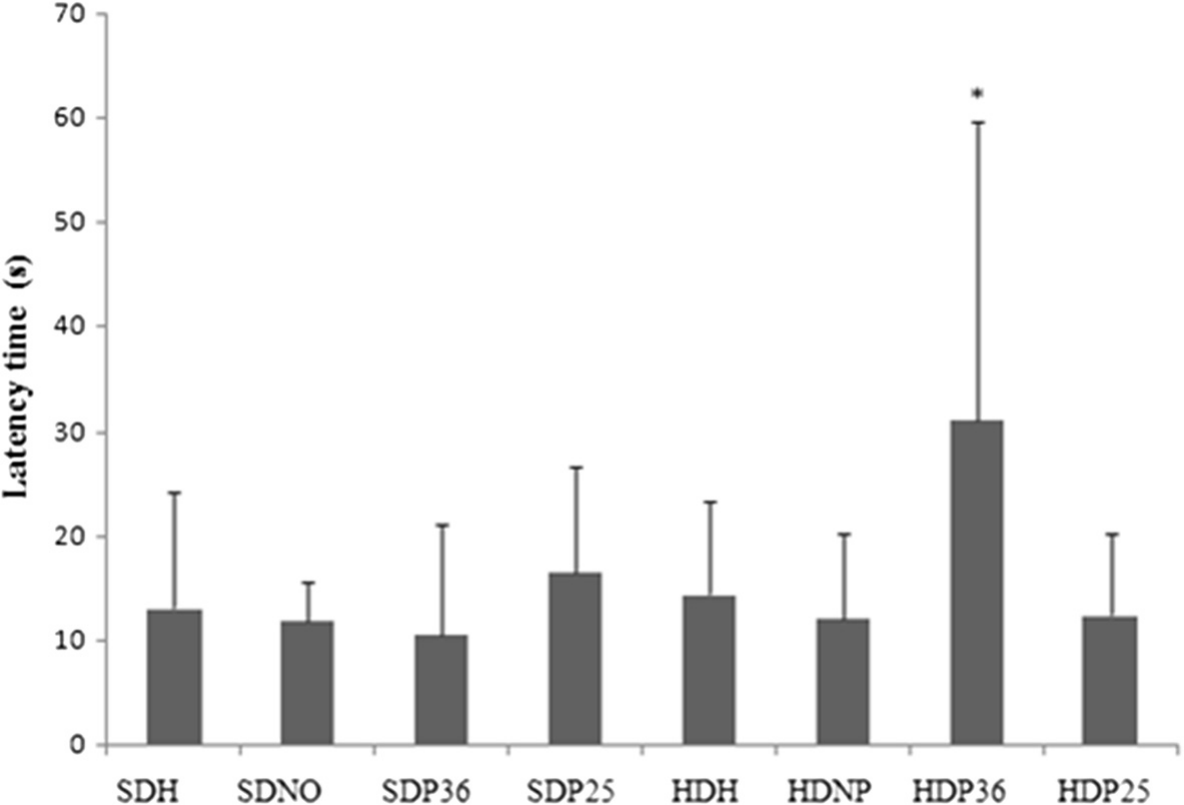
Alimentary behavior test

**Fig. 4:**
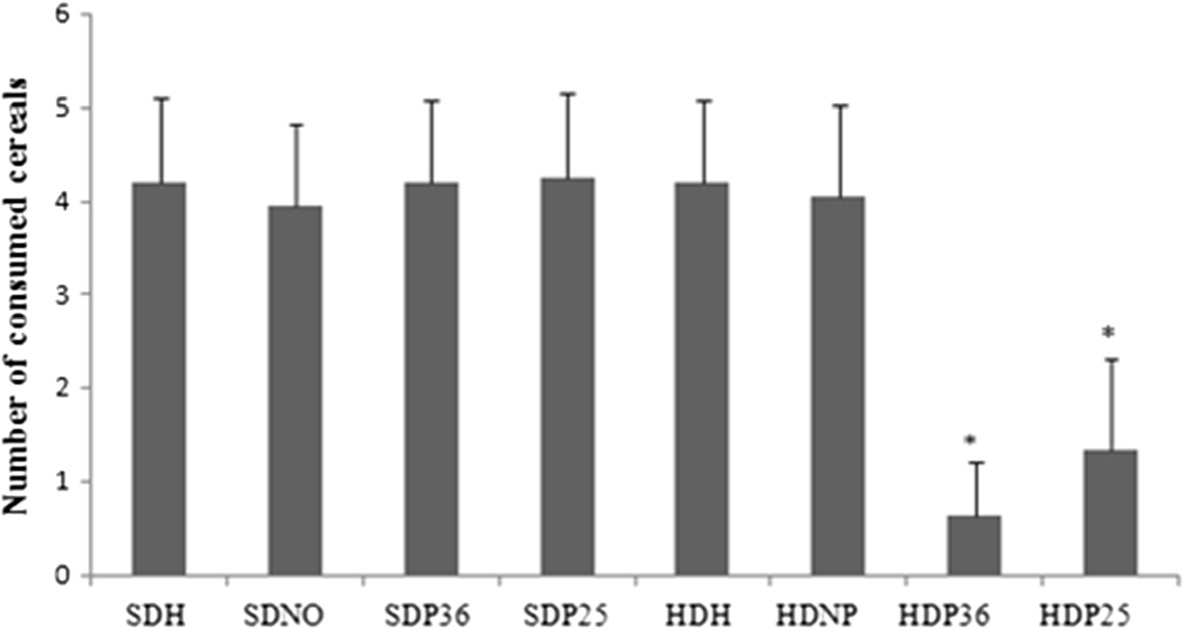
Alimentary behavior test. *Significant (*p* < 0.001) difference of subgroups HDP36 and HDP25 compared to the others

No significant difference was found between the groups studied in alimentary behavior regarding latency to feed (Fig. 3), i.e., both groups reached the food, on average, within the same timeframe. Nevertheless, the number of cereal bits consumed was significantly lower (*p* < 0.001) in subgroups HDP36 and HDP25 compared to HDH and HDNP (Fig. 4).

Based on the data obtained, the points assessed impact the motivation for food consumption, according to the alimentary behavior test. Although latency to feed did not differ between the groups, the group treated with pharmacopuncture consumed fewer cereal bits, which suggests the treatment resulted in lower appetitive behavior in the animals treated with the specific points.

### Elevated plus maze

**Fig. 5:**
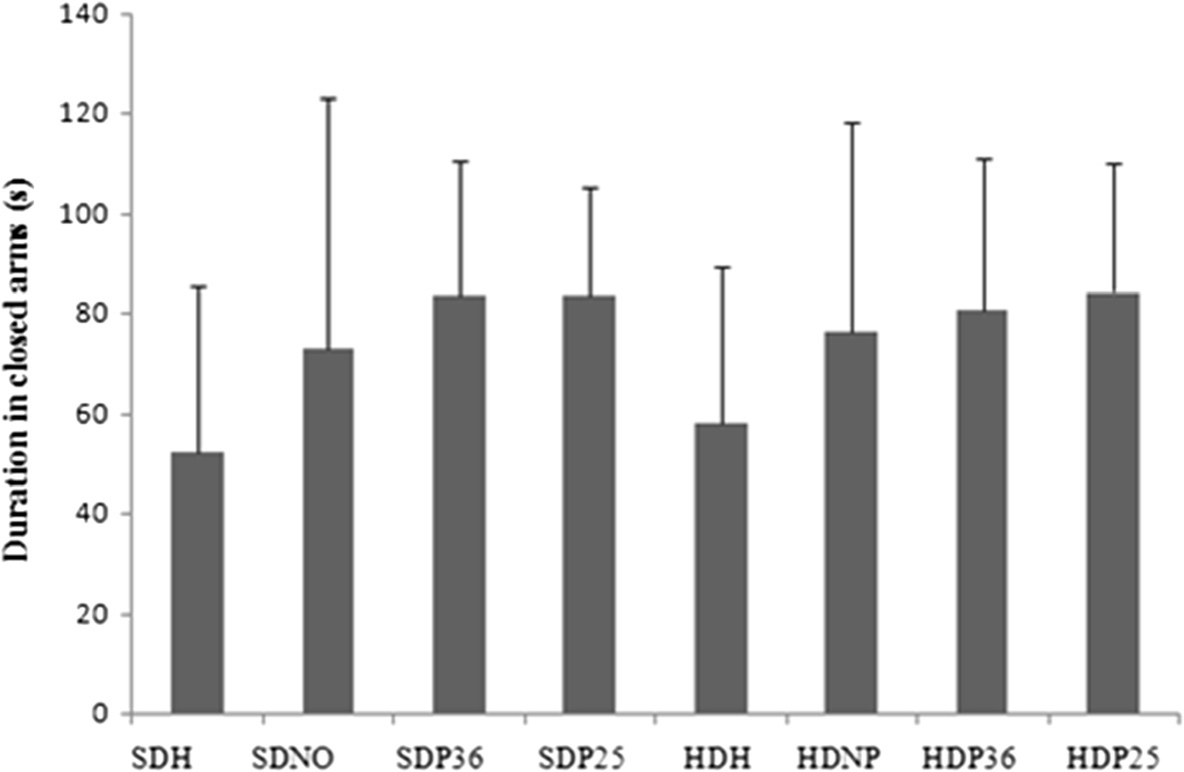
Elevated Plus Maze test. Time in the open-sided arm

The elevated plus maze test did not show a significant difference among the groups analyzed (Fig. 5). The present study suggests that points ST 36 and ST 25 did not impact the animals’ anxiety, as shown by the elevated plus maze test. Anxiety is mentioned as a factor that favors food intake [3], however, no significant difference in the elevated plus maze test was found that shows an effect of this component.

## Visceral fat weight

**Fig. 6:**
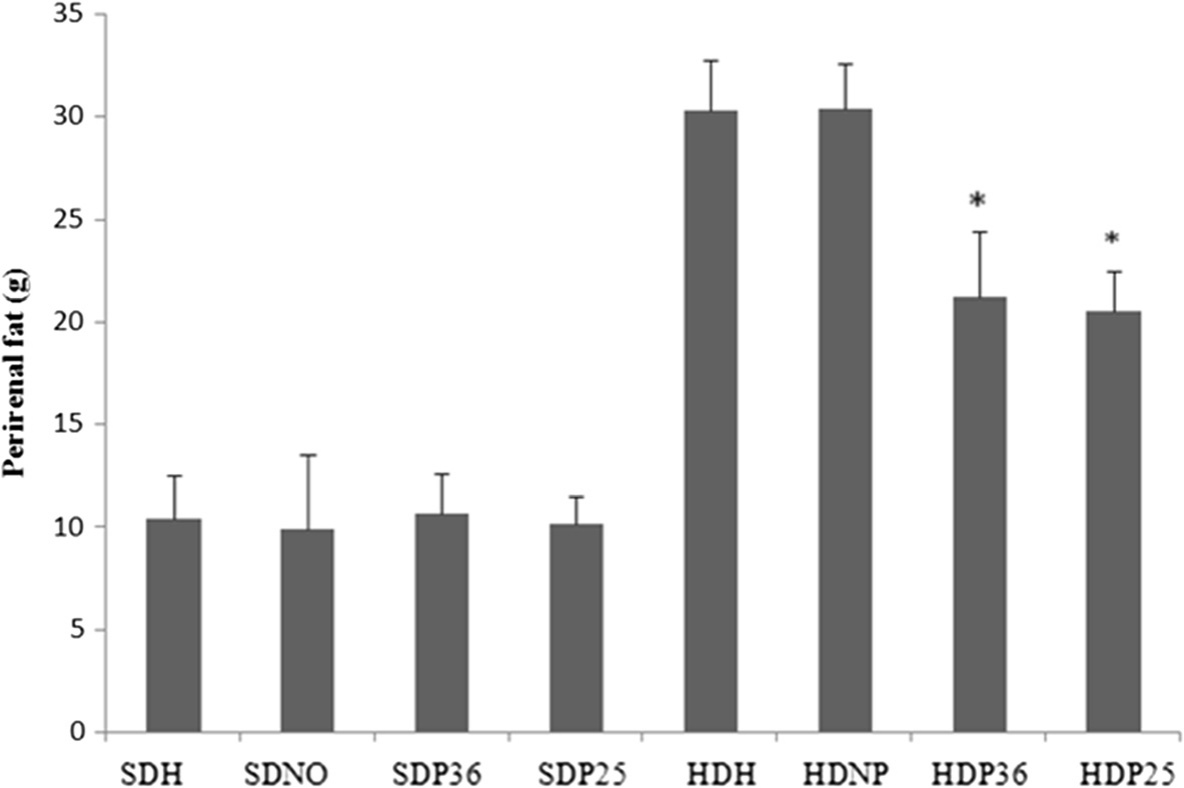
Post mortem perirenal fat. *Significant (*p* < 0.001) difference when compared to subgroups HDH and HDNP

**Fig. 7:**
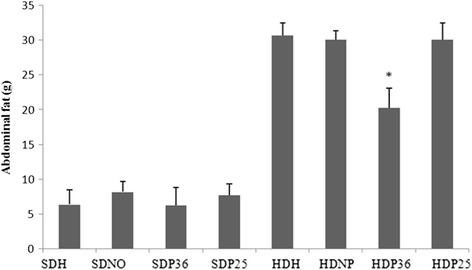
Post mortem abdominal fat. *Significant (*p* < 0.001) difference when compared to subgroups HDP25, HDH, and HDNP

The subgroups fed the standard diet had significantly less (*p* < 0.001) perirenal and abdominal fat compared to the subgroups that were fed the hypercaloric diet. Likewise, less perirenal fat (*p* < 0.001) was found in subgroups HDP36 and HDP25 compared to HDH and HDNP, which shows an effect of acupuncture in body weight accumulation (Fig. 6). At the same time, only subgroup HDP36 had significantly (*p* < 0.001) lower abdominal fat weight compared to HDH and HDNP (Fig. 7).

A reduction in perirenal and abdominal fat was also observed. This also matches the experiments carried out with electroacupuncture in point ST 36 in rats [15]. The perirenal fat reduction data obtained in the present study are promising, since inner fat, which is harder to be reduced exclusively through dietary restriction [3], could be reduced using specific acupuncture points.

## Biochemical analysis

**Fig. 8:**
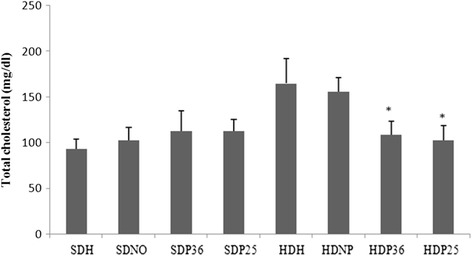
Plasma cholesterol. *Significant (*p* < 0.001) difference when compared to subgroups HDH and HDNP

**Fig. 9:**
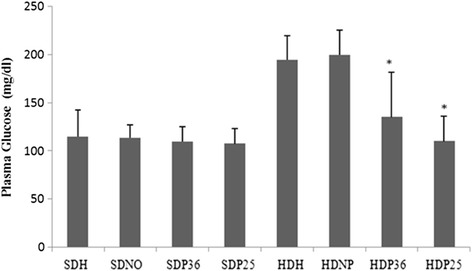
Plasma glucose. *Significant (*p* < 0.001) difference when compared to subgroups HDH and HDNP

Significant differences were found in the levels of glucose and plasma cholesterol when the four subgroups that were fed a hypercaloric diet were compared amongst themselves. However, no significant difference was found when compairing the four subgroups that were fed standard diet. Subgroups HDH36 and HDH25 had significantly (*p* < 0.001) lower glucose and cholesterol levels compared to the ones that were also fed the hypercaloric diet with no pharmacopuncture intervention (HDH and HDNP). The reduction in glucose and cholesterol levels in subgroups HDH36 and HDH25 was so significant that these animals did not significantly differ from the ones that were fed the standard diet (SDM, SDNP, SDP36, and SDP25) (Figs. 8 and 9).

**Fig. 10:**
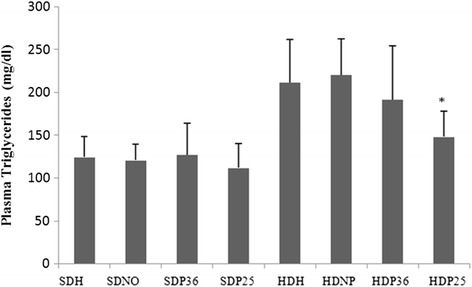
Plasma triglycerides. *Significant (*p* < 0.001) difference when compared to subgroups HDH and HDNP

Regarding triglycerides, only subgroup HDP25 had a significant (*p* < 0.001) reduction in plasma levels compared to the other ones fed the hypercaloric diet (HDH, HDNP, and HDP6). No significant difference was found when subgroup HDP25 was compared with the ones fed the standard diet (SDM, SDNP, SDP36, and SDP25) (Fig. 10).

It was also observed that using pharmacopuncture in points ST 36 and ST 25 significantly lowered glucose and cholesterol levels, and that ST 25 decreased triglyceride levels when compared to the control group.

Yang et al. [32] also observed the same in the treatment with electroacupuncture in these same points in rats, Wang et al. [30] in Sprague–Dawley rats, and Lee et al. [18] in male Wistar rats. On the other hand, Tian et al. [29] did not find differences in plasma glucose and triglyceride levels of Sprague–Dawley rats when using electroacupuncture in point ST 36.

In conclusion, the results hereby presented from the use of pharmacopuncture in obese Wistar rats show that this technique may likely be recommended as a complement in the treatment of patients with high cholesterol, glucose, and triglyceride levels.

## Conclusions

The present study allows a conclusion that pharmacopuncture in points ST 36 and ST 25 yields favorable results in reducing body weight, visceral fat, and biochemical parameters such as cholesterol, triglycerides, and glucose in obese rats. Likewise, it decreases appetitive behavior as shown by the alimentary behavior test.
